# MUC1-Targeted Cancer Cell Photothermal Ablation Using Bioinspired Gold Nanorods

**DOI:** 10.1371/journal.pone.0128756

**Published:** 2015-07-06

**Authors:** Daria C. Zelasko-Leon, Christina M. Fuentes, Phillip B. Messersmith

**Affiliations:** 1 Department of Biomedical Engineering, Chemistry of Life Processes Institute, Northwestern University, Evanston, Illinois, United States of America; 2 Department of Bioengineering, University of California, Berkeley, Berkeley, California, United States of America; 3 Department of Materials Science and Engineering, University of California, Berkeley, Berkeley, California, United States of America; Brandeis University, UNITED STATES

## Abstract

Recent studies have highlighted the overexpression of mucin 1 (MUC1) in various epithelial carcinomas and its role in tumorigenesis. These mucins present a novel targeting opportunity for nanoparticle-mediated photothermal cancer treatments due to their unique antenna-like extracellular extension. In this study, MUC1 antibodies and albumin were immobilized onto the surface of gold nanorods using a “primer” of polydopamine (PD), a molecular mimic of catechol- and amine-rich mussel adhesive proteins. PD forms an adhesive platform for the deposition of albumin and MUC1 antibodies, achieving a surface that is stable, bioinert and biofunctional. Two-photon luminescence confocal and darkfield scattering imaging revealed targeting of MUC1-BSA-PD-NRs to MUC1^+^ MCF-7 breast cancer and SCC-15 squamous cell carcinoma cells lines. Treated cells were exposed to a laser encompassing the near-infrared AuNR longitudinal surface plasmon and assessed for photothermal ablation. MUC1-BSA-PD-NRs substantially decreased cell viability in photoirradiated MCF-7 cell lines vs. MUC1- MDA-MB-231 breast cancer cells (p < 0.005). Agents exhibited no cytotoxicity in the absence of photothermal treatment. The facile nature of the coating method, combined with targeting and photoablation efficacy, are attractive features of these candidate cancer nanotherapeutics.

## Introduction

Au nanorods (AuNRs) are highly attractive constructs for tumor therapy due to their ease of synthesis, tunable near-infrared (NIR) localized surface plasmon resonance (LSPR), and large, functionalizable surface areas [1, 2]. The LSPR enhances optical properties, giving rise to high absorbance, scattering, and two-photon luminescence phenomena that can be exploited for photothermal cancer therapy and diagnostic imaging [1, 2, 3]. Patients with poor tumor margins or microscopic disease may be poor candidates for invasive surgical procedures and stand to benefit from improved multimodal approaches [1]. The non-invasive penetration of NIR energy to AuNR-treated tissues permits localized hyperthermia resulting in tumor ablation. As an added benefit, this heating may enhance tissue perfusion, increasing subsequent nanoparticle loading and the efficacy of adjuvant chemotherapy or radiation [4, 5].

A primary challenge in AuNR-based therapies relates to modifying or replacing the initial CTAB bilayer with a surface coating that is both bioinert and biofunctional [6]. A variety of passivation strategies have been developed to overcome this problem. These strategies include modification with amphiphillic synthetic polymers such as poly(ethylene glycol)-thiols (PEG-SH)[7], lipids [8], electrostatic layers of polyanionic and polycationic polymers [9], and most recently, proteins [10, 11]. The observed development of a protein corona following nanoparticle contact in serum-containing biological media has supported interest in albumin as a potential nanoparticle passivation agent [11].

Transmembrane mucins rich in glycosylated proline, threonine, and serine domains span the epithelial cell membrane and provide a barrier function through ectodomains that project over 100 nm from the cell surface [12]. Autoproteolysis generates C-terminal (MUC1-C) and N-terminal (MUC1-N) subunits, the latter of which is anchored to the cell surface through a stable but non-covalent complex with MUC1-C. While mucins are typically expressed at the apical surface of cells to protect against environmental toxins, chronic stress induces a loss in cell polarity leading to interactions of mucins with basolateral surface signaling molecules such as receptor tyrosine kinases, triggering the downstream activation of proliferation and survival genes. MUC1 is upregulated in response to the influx of inflammatory cytokines during inflammation and infection, leading to loss of polarity and strengthening the protective function of the mucosal barrier.

Although transient MUC1 activity functions to reduce inflammation, long-term overexpression promotes aggressive phenotypes in human cancers. MUC1-N contains heavily glycoslyated tandem repeats of 20 amino acids and is aberrantly underglycosylated in epithelial carcinomas, exposing residues implicated in the immunosurveillance of cancer [13]. MUC1-N is capable of blocking surface interactions and can also undergo secretion from the cell membrane, permitting the receptor-like activation of MUC1-C in a variety of tumor signaling pathways [12]. The significance of MUC1 as a relevant therapeutic target is highlighted by its atypical expression in > 64% of carcinomas diagnosed annually, and in over 90% of breast carcinomas irrespective of hormone or growth factor receptor status [14].

The function and exploitation of MUC1 as a tumor antigen continues to be elucidated [15]. Despite its antenna-like physical manifestation that in principle lends well to therapeutic targeting of mucin-expressing tumors [12, 16], MUC1 has been underexploited in targeted cancer therapy [17, 18, 19, 20]. Studies have demonstrated that MUC1 overexpression directly promotes *in vivo* transformation of the mammary gland and its aberrant expression in transformed cells can induce steric blocking or activation of cell surface receptors, serving as a driving force for invasion [21] and chemotherapy resistance [22, 23]. Clinically, detection of circulating serum mucin levels is an FDA-approved prognostic factor in the treatment of breast cancer [12] and phase III clinical trials evaluating MUC1 based immunotherapies are underway [24].

**Fig 1:**
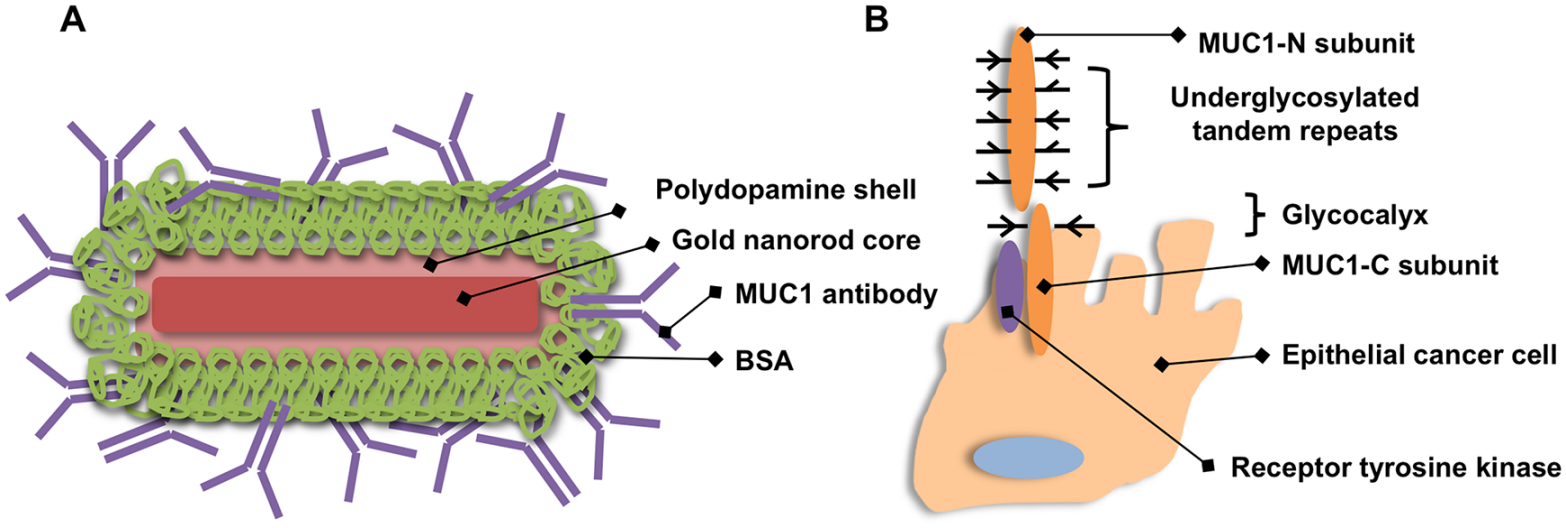
Schematic illustration of MUC1 antibody-conjugated gold NRs (anti-MUC1-BSA-PD-NRs) with a polydopamine adlayer and BSA coating. MUC1 antibodies serve as novel targeting constructs in the application of plasmonic photothermal therapy (A). Probing MUC1 targeting at underglycosylated N- and C-terminal domains in various epithelial carcinomas will expand our cancer-targeting repertoire with the potential for synergistic therapeutic effects (B, adapted from [12]).

Here, we demonstrate the polydopamine-mediated (PD) conjugation of gold nanorods (AuNRs) with bovine serum albumin (BSA) and mucin 1 monoclonal antibodies (anti-MUC1) for passivation and targeting, respectively (Fig 1). The major goals of this work were to identify optimal conditions for nanoparticle functionalization and to demonstrate the feasibility of photothermal ablation of MUC1 positive cancer cells via biofunctionalized AuNRs. The results establish optimal conditions for surface modification of AuNRs with BSA and MUC1 antibody and physiologic stability. Successful targeting and photoablation of MUC1 positive cancer cells suggests these constructs may be useful anticancer therapeutics in the future.

## Materials and Methods

### Materials

Dopamine hydrochloride, cetyltrimethylammonium bromide (CTAB, 99%), sodium tetrachloroaurate (III) dihydrate (NaAuCl_4_ · 2H_2_O, 99%), sodium borohydride (NaBH_4_, 98%), ascorbic acid, glycine, and silver nitrate (AgNO_3_, 99%) were utilized for AuNR synthesis. The pH value of the glycine solution (0.2 M) was adjusted to 8.0 with 2 M sodium hydroxide before use. All reagents were obtained from Sigma-Aldrich (St. Louis, MO) unless otherwise noted. AlexaFluor 633 goat anti-mouse immunoglobulin (IgG) was purchased from Invitrogen (Carlsbad, CA). Anti-MUC1-N (VU4H5), anti-MUC1-N-PE (VU4H5 PE), mouse anti-MUC1-C (H-6), goat anti-mouse-HRP IgG, goat anti-rabbit-HRP IgG, and rabbit anti-BSA (B-140) primary antibodies were purchased from Santa Cruz Biotechnology (Santa Cruz, CA). Goat anti-mouse IgG and goat anti-rabbit IgG antibodies conjugated to PE and/or HRP were purchased from Santa Cruz Biotechnology (Santa Cruz, CA). Ultrapure, deionized water (18.2MΩ · cm) was used to prepare all aqueous solutions. SCC15, MCF-7 and MDA-MB-231 cancer cell lines were generous gifts from the laboratories of Dr. David Crowe at the University of Illinois—Chicago and Dr. Dean Ho at Northwestern University.

### Methods

#### Synthesis of gold NRs

The synthesis of CTAB-coated AuNRs (CTAB-NRs) is well established in the literature and was performed according to a slightly modified method utilized by Huang and coworkers [2]. All reagents were procured from Sigma Aldrich (St. Louis, MO) unless otherwise noted. A 0.2 M CTAB aqueous solution (5.0 mL) was heated to 30°C and mixed with 0.5 mM NaAuCl_4_ (5.0 mL). Ice-cold 0.01 M NaBH_4_ (0.6 mL) was added to this mixture and sonicated for 5 minutes until a yellow-brown seed solution developed. Next, 50.0 mL of 0.2 M CTAB was gently mixed with 50.0 mL 1.0 mM NaAuCl_4_ and 0.1 mL 0.1 M AgNO_3_ to form a growth solution. Ascorbic acid (78.8 mM, 0.7 mL) was added to the growth solution as a mild reductant, followed by addition of 120 μL of the seed solution. After 45 minutes, 100 mL of this AuNR solution was mixed with 100 mL 0.2 M glycine (pH 8.0). This solution was allowed to react overnight without stirring at ambient temperature.

#### Biofunctionalization of gold NRs

For the synthesis of BSA-PD-NRs, 1 mL aliquots of CTAB-NRs were centrifuged at 10,360 *x g* for 10 min to form a pellet. The supernatant was discarded and the pellet was redispersed in 1.0 mL 10 mM bicine buffer (pH 8.5) supplemented with 0.1 mg/mL dopamine hydrochloride. A polydopamine coating was developed with sonication at 37°C for 30 minutes. A 1.0 mL solution of 20 mg/mL BSA prepared in PBS (Ca^2+^ and Mg^2+^ free) was added to this PD-NR mixture and allowed to react for another 30 minutes with sonication. Following overnight reaction with gentle agitation, samples were centrifuged, redispersed in water or PBS, and stored at room temperature until further use. For antibody conjugations, 0.25–5.0 μg antibody was either reacted directly with CTAB- or PD-NRs prior to BSA addition or premixed with BSA or buffer solutions prior to addition.

#### Antibody binding and quantification

*Enzyme-linked immunosorbent assay (ELISA)*: Goat anti-mouse IgG coated 96-well ELISA microplates (Pierce Biotechnology, Rockford, IL) were rinsed with PBS + 0.05% Tween-20 (PBST). Mouse anti-MUC1-N/C antibody standards and AuNR supernatant samples containing unknown quantities of MUC1-N or -C (100 μL/well) were loaded and allowed to capture for 1 hr at room temperature with gentle mixing. Plates were washed 3X in 200 μL PBST. Secondary goat anti-mouse IgG-HRP (Santa Cruz Biotechnology, 100 uL/well, 1:6000 dilution) was incubated for 1 hour at 37°C with mixing. The plate was washed 3X with PBST and a 1 mg/mL 2,2'-azino-bis(3-ethylbenz-thiazoline-6-sulfuric acid) (ABTS) solution was prepared in 50 mM citric acid and 100 mM dibasic sodium phosphate. Immediately prior to use, the ABTS solution was mixed with 10 μL 30% H_2_O_2_ and added to wells (100 μL). ABTS color development based on the presence of HRP-tagged secondary antibody was measured at 405 nm within 15 minutes on a Synergy H4 Hybrid Multi-Mode Microplate Reader (BioTek, Winooski, VT). The quantity of AuNR-bound MUC1-N and MUC1-C antibodies was determined by subtracting the concentrations detected in the sample supernatants from the known loading conditions during preparation. Concentrations were calculated from point-to-point linear regression standard curves.

#### Optical spectroscopy

*UV-Vis-NIR spectrophotometry*: UV-Vis-NIR spectra of variously modified AuNRs were recorded in a Hitachi U-2010 spectrophotometer (Hitachi City, Japan). Longitudinal LSPR peak positions were determined and used as an indicator of surface modification and aggregation.

*Circular dichroism*: Circular dichroism (CD) spectra were recorded with a J-815 CD Spectrophotometer (Jasco, Easton, MD) in the far-UV range (190–300 nm, 1 nm resolution) in 1 mm pathlength quartz cuvettes at ambient temperature. AuNR pellets (30 μL) were diluted in 900 μL ultrapure water for all measurements. BSA (0.25 mg/mL) served as a protein reference while ultrapure water was used for background subtraction. The spatial arrangement and backbone conformation of protein amides gives rise to the characteristic CD spectra of specific secondary structures. Minima at 222 and 208 nm result from α-helical protein amides and light polarized in the direction of α-helices, respectively [25]. While methods for the calculation of % helicity are widely available [26], they require protein concentrations that could not be precisely determined in our experiments due to catecholamine interference [27]. Thus, to quantify the extent of secondary structure preservation in BSA, the α-helical propensity [26],

$$\text{p}\left(\alpha \right)=\frac{\theta_{222}}{\theta_{208}}$$ (1)

can be used as a coarse but convenient estimate of α-helical content, where *θ* represents the raw ellipticity in millidegrees at 208 and 222 nm, respectively. Ratios between 0.80 and 0.95 represent single chain α-helices and increase with increasing α-helical content [26].

#### Cell culture

MCF-7 breast cancer cells were grown in high glucose DMEM (Invitrogen, Carlsbad, CA) supplemented with 10% FBS (Invitrogen), 1% gentamicin sulfate (Invitrogen), and 10 μg/mL recombinant human insulin (Santa Cruz Biotechnology). MDA-MB-231 cells were cultivated in RPMI 1640 (Invitrogen) with 10% FBS and 1% gentamicin sulfate. SCC15 oral squamous cell carcinoma was grown in high glucose DMEM supplemented with 10% FBS and 1% gentamicin sulfate. All cell lines were grown at 37°C in humidified incubators with 5% CO_2_.

#### Cell imaging

For fluorescence and two-photon luminescence (TPL) confocal imaging, suspensions of cells (1x10^5^) in 0.2 mL media were seeded into each of the 8 wells of a Lab-Tek chambered coverglass slide (Nunc, Rochester, NY). Cells were grown to confluence and incubated with fresh media containing AuNRs (3 pM). Cells were incubated with AuNRs for 24 hours, rinsed twice with PBS, and imaged live for TPL emissions in fresh media. Confocal images were acquired with an inverted Axio Observer. Z1 microscope equipped with a 20X objective lens and heated stage (37°C) set for 5% CO_2_ incubation (Zeiss, Oberkochen, Germany). For TPL imaging, AuNRs were excited by a Mai Tai femtosecond (fs) Ti:Sapphire laser (Mai Tai, Spectra-Physics, Santa Clara, CA) with a pulse rate of 130 fs and a repetition rate of 80 MHz. The TPL excitation wavelength was adjusted to the LSPR peak of the AuNR sample (780 nm). An internal spectral detector adjusted for emissions between 500–615 nm detected the TPL from AuNRs. A 543 nm HeNe laser was used for brightfield imaging. In darkfield (DF) scattering experiments, cells were seeded (1x10^4^ cells/well) on 16 well chamber slides (Lab-Tek, Nunc), grown to confluence, and treated with 3.75 pM AuNRs in media for 1 hour. Individual wells were rinsed 3X with PBS. The wells and chamber gaskets were carefully removed and the slide was coverslipped for immediate imaging at 40X magnification with an upright Leica DM2500 (Wetzlar, Germany) darkfield microscope. Images were acquired at a constant exposure (f-stop 1/15) via an EOS Rebel T2i camera.

#### Electron microscopy

Electron microscopy (EM) grids (Ted Pella) were loaded with pelleted AuNRs (5 μl, 1 nM), counterstained with phosphotungstinic acid, and dried overnight at ambient conditions. Grids were imaged with a Hitachi HD-2300 Ultra High Resolution field-emission scanning transmission electron microscope (FE-STEM) (Hitachi City, Japan) in transmission EM (TEM) and secondary electron (SE) modes.

#### ζ-Potential analysis

Modified and unmodified AuNRs (15 pM) were injected into standard folded capillary cells for ζ-potential measurements. Measurements were performed on a Zetasizer Nano (Malvern Instruments, Worcestershire, United Kingdom) at ambient temperature and calculated as the mean of seven scans.

#### Photothermal therapy with MUC1-modified AuNRs

MCF-7, MDA-MB-231, and SCC15 (5.0 x 10^5^) cancer cells were cultured in 12 well TCP plates and grown to confluence over 2–3 days. Wells were washed with PBS and treated with 0–3 pM anti-MUC1-N, anti-MUC1-C, or BSA-only-modified AuNRs diluted in cell culture media. Following 1 h incubation at 37°C and 5% CO_2_, wells were rinsed 3X with PBS to remove unbound AuNRs and marked at two focal points with equivalent confluence levels. One of each of these points was exposed to a SuperK Versa broadband laser source (NKT Photonics, 480–850 nm, 1 mm spot) for 3.5 min at a power density of 0.18 W/nm at the plasmon center wavelength (780 nm). After a 24 hr incubation, cells were rinsed with PBS, and stained with calcein AM (2 uM) and propidium iodide (4 uM) for live/dead imaging. Fluorescence microscopy was performed with a Leica DMIRB microscope (Wetzlar, Germany) equipped with a 250 W Hg arc lamp and a QIClick camera (QImaging, Surrey, BC Canada). Points of irradiation and non-irradiation were captured in each well for comparison. Images were processed with the GNU Image Manipulation Program (GIMP, v. 2.8.0). ImageJ was used to convert live/dead images into black (background) and white (cells) thresholded masks. The area percentage of background pixels with (Area %_+NIR_) or without laser exposure (Area %_-NIR_) was calculated with the *Measure* function in ImageJ. The following equation was used to quantify treatment efficacy:

$$\text{\% Viable Area}=\frac{100-Area\;{\%}_{+NIR}}{100-Area\;{\%}_{-NIR}}\times \;100$$ (2)

Results were normalized to untreated wells ± NIR exposure. Experiments were repeated with SCC15 oral and MDA-MB-231 breast cancer cell lines.

#### Statistical analysis

Student’s t-test was used to analyze percent antibody loading via ELISA. Two-way analysis of variance (ANOVA) with Bonferroni post-tests was used to determine whether the mean % viable areas among MUC1^+^ and MUC1^-^ cell lines were significant with respect to dosing and treatment conditions. The differences were considered statistically significant when the *P* value was < 0.05 (n = 3–6). All analyses were carried out by GraphPad Prism version 5.0a for Mac (GraphPad Software, La Jolla, CA).

## Results and Discussion

### Albumin as a surface passivating agent

Minimal nonspecific fouling of surfaces is often a desirable goal in biomedical research and medical device design [28, 29], for which the grafting of biomolecules and synthetic polymers to a surface has been proposed as a solution [30, 31]. The degree and nature of nonspecific protein adsorption to a surface is dependent upon many factors including protein composition (size, concentration, charge, and internal stability), environmental conditions (pH, temperature, and ionic strength), and surface properties (morphology, charge, and free energy) [32]. The use of adsorbed BSA as an approach to surface passivation has long been an intriguing strategy for biomedical researchers. One of the earliest uses of this strategy was the observation by Packham and others that platelets resist adsorption to albumin-coated glass tubes [33]. Passivation with albumin was proposed for plasmapheresis circuits, arterial prosthetics, and other blood-contacting devices to prevent thrombogenesis, however this approach showed limited efficacy due to low surface coverage or displacement of physisorbed albumin by proteins with higher binding affinities [34].

Subsequent attempts were made to cross-link adsorbed albumins with glutaraldehyde or gamma irradiation treatments; however, the resultant loss in protein flexibility limits their ability to contribute to steric repulsion [35]. Since these first observations, albumin has been used to modify iron oxide [36], polystyrene [37], Ag and Au [38, 39], quantum dot [40], and liposomal nanoparticles [41]. Studies have not only demonstrated that BSA-conjugated nanoparticles reduce aggregate formation, but they have also revealed enhancement of quantum yield, which is of relevance to the photothermal applications explored in this study [42].

### Polydopamine for functionalizing nanoparticles with biomolecules

In this study we describe a mussel adhesive protein inspired strategy for anchoring BSA and antibody onto the surface of gold nanorods. The marine mussel’s promiscuous fouling of organic and inorganic surfaces has been attributed by Waite and others to the unusual proteins found in the terminal adhesive plaques of its byssal threads [43, 44]. Specifically, high levels of the catechol-containing amino acid 3-4-dihydroxyphenylalanine (DOPA) and the amine-containing amino acid lysine occur in byssal plaques, and this observation has greatly influenced the design of versatile molecular adhesives, anchors for synthetic polymers, and general strategies for surface coatings [44, 45].

Motivated by the high catecholamine content of mussel adhesive proteins, dopamine was identified as a simple structural mimic of the mussel foot protein 5 (Mfp-5) and shown to spontaneously deposit thin melanin-like films on virtually any bulk material surface [46]. Polydopamine (PD) films are formed through the spontaneous polymerization of dopamine molecules under mildly alkaline aqueous conditions that mimic the marine environment. Although the mechanism of formation and final composition of PD are still under investigation [47, 48, 49, 50, 51, 52], under the conditions normally used to form PD coatings dopamine is oxidized to yield dopamine-quinone which subsequent to intramolecular rearrangement, further oxidation and intermolecular coupling, yields a eumelanin-like heterogeneous polymer [44]. It is likely that a variety of structural subunits and bonding types are found within the PD film as well as at the PD-substrate and PD-protein interfaces, including covalent bonds, electrostatic and strong noncovalent interactions such as charge transfer, hydrogen bonding, and π-stacking. The adhesive and reactive nature of polydopamine thin films was exploited as a convenient platform or ‘primer’ onto which can be deposited additional secondary coatings via a variety of potential mechanisms, including covalent linkage with organic thiol, amine, and histidine residues [53]. With this two-step approach a wide variety of functional applications of polydopamine have been developed for biomedical applications [54], such as the grafting of PEG-thiols to suppress biofouling [46, 55] or the specific promotion of cell adhesion to surfaces [46].

### Preparation and characterization of BSA modified AuNRs

**Fig 2:**
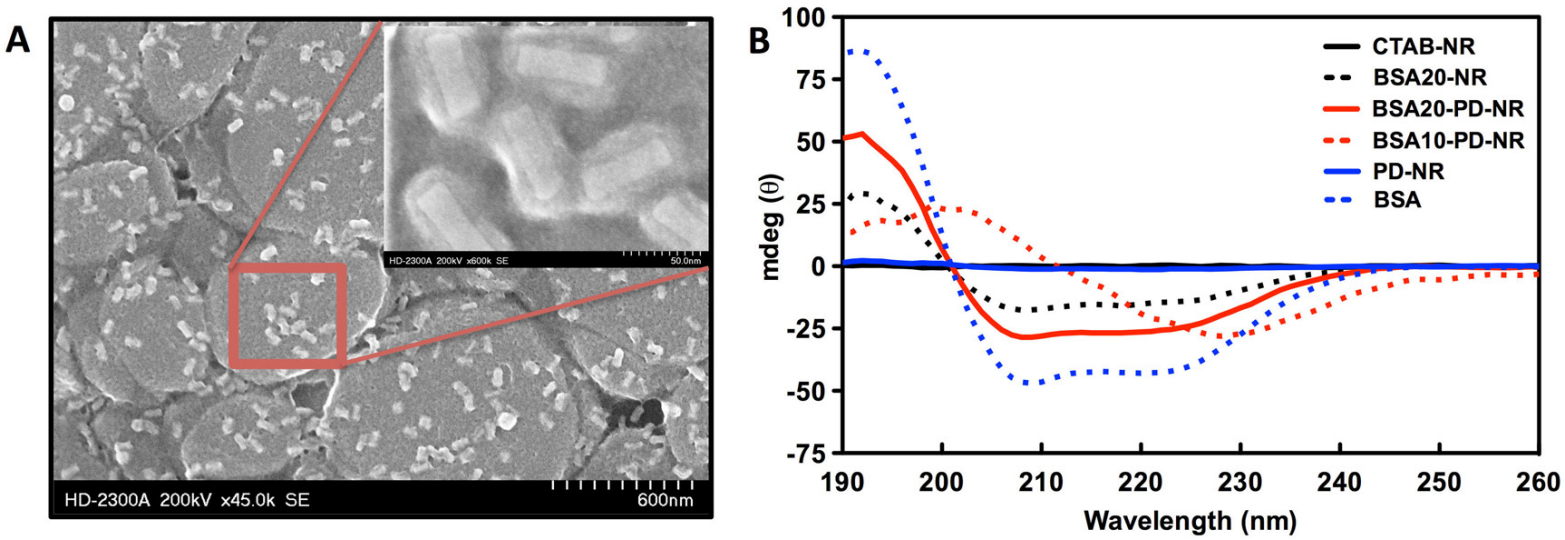
Bovine serum albumin (BSA) coating on polydopamine-primed gold NRs. (A) Electron microscopy of BSA-PD-NRs in secondary electron mode. Scale bar = 600 nm; Inset = 50 nm; (B) Circular dichroism of modified vs. unmodified gold NRs. BSA-modified NRs were modified with 10 or 20 mg/mL BSA, as indicated. The concentration of the BSA control was 0.25 mg/mL. Protein denaturation into β-sheet formation is indicated on PD-treated NRs modified with a sub-optimal concentration of BSA. Otherwise, BSA secondary structure is preserved, as quantified by the respective α-helical propensity of each modification.

Modification of AuNRs with PD was performed as described previously [56, 57], yielding AuNRs surrounded by a thin PD coating. The presence of PD was confirmed by UV-vis red shift (Fig A in S1 File). PD-NRs were found to aggregate in buffer, but redispersion in BSA solution and isolation by centrifugation led to stable dispersions of BSA-PD-NRs. Using the solution AuNR LSPR intensity and peak shift (Fig B in S1 File) analyzed via UV-Vis-NIR spectrophotometry as a measure of NR stability, screening of a range of BSA concentrations (0–30 mg/mL) used in the two-step coating method led to the identification of an optimal protocol consisting of 0.1 mg/mL dopamine for PD coating of CTAB-NRs followed by stabilization in ≥10 mg/ml BSA. Under these optimal conditions, aggregation of BSA-PD-NRs was seven-fold less compared to PD-NRs (Fig B in S1 File). All subsequent experiments were performed on PD-NRs modified at 20 mg/mL BSA in order to ensure full passivation of the PD underlayer that may otherwise serve to promote AuNR aggregation. AuNRs (20 x 60 nm) modified with PD and BSA (20 mg/mL) were imaged via TEM following counterstaining with phosphotungstic acid to provide contrast for the organic BSA layer. A layer measuring ~15 nm in thickness was visualized on well-separated BSA-PD-NRs in SE mode (Fig 2A). The attenuation of XPS Au4f signal of a gold surface observed upon exposure to dopamine and BSA is consistent with the deposition of a PD coating followed by grafting of BSA (Fig C in S1 File).

AuNRs were further evaluated via UV-Vis-NIR spectroscopy to analyze changes in the longitudinal LSPR peak position (Fig B in S1 File). BSA-DP-NRs demonstrated a plasmon red shift of 10 nm due to changes in the local dielectric environment resulting from the surface adsorption events. This change in the LSPR wavelength maximum, Δλ_max_, corresponds to an adsorbed thickness *d* of 15 nm based on equation [57, 58],

$$d=-\text{ln }\left(\frac{\Delta \lambda_{max}}{m\Delta n}-1\right)*\frac{l_{d}}{2}$$ (3)

Where *m* is the bulk refractive index response of the nanoparticle, Δ*n* is the refractive index change induced by the adsorbing species, and *l*_*d*_ is the decay length of the electromagnetic field. This calculated thickness is in agreement with our TEM observations (Fig 2A). Additionally, ζ-potential measurements were recorded to track AuNR biofunctionalization and are reported in Table 1. The stepwise change in ζ-potential with PD and BSA modification culminated in a negatively-charged BSA coating.

**Table 1:** AuNR zeta-potential measurements.

Sample	ζ-potential (mV)
CTAB-NR	20.0 ± 0.7
PD-NR	2.6 ± 0.6
BSA-NR	-21.7 ± 1.3
BSA-PD-NR	-30.3 ± 0.6
IgG-BSA-PD-NR*	-14.9 ±1.2

Decreasing zeta-potential measurements verify successive surface modifications.

*IgG—AlexaFluor 633 goat anti-mouse immunoglobulin.

BSA binding to PD-NRs most likely occurred via the noncovalent mechanisms described above or by covalent coupling to any of 60 available surface lysine residues [38]. Parallel experiments conducted on untreated CTAB-NRs dispersed in 10 mg/ml BSA did not yield a stable suspension, indicating the importance of PD in enhancing BSA passivation. Although higher concentrations of BSA were found to stabilize CTAB-AuNRs in the absence of PD as indicated by sedimentation experiments (Fig B in S1 File) and ELISA (data not shown), these constructs failed to facilitate optimal antibody immobilization in later experiments.

The tertiary structure of serum albumin is composed of three domains (I-III), which are further organized into nine loops by 17 disulfide linkages [59]. Notably, BSA maintains a single free sulfhydryl residue at Cys-34, whose presence is implicated in both metal chelation and free radical scavenging activity [60]. In physiological conditions, BSA maintains 67% α-helical content, 10% β-turns, and 23% extended chains [59, 61]. At physiologic pH BSA maintains a net negative charge (pI = 4.7), contributing to its excellent water solubility, although a decrease in effective charge is expected as a function of increasing solution pH [59, 62], elucidating the potential importance of electrostatic interactions on albumin binding to CTAB or polydopamine modified surfaces. Importantly, the electrostatically driven adsorption of negatively charged BSA at pH 8.5 to AuNR surfaces decorated by cationic CTAB ammonium head groups was found to induce some denaturation of α-helical secondary structure as observed via CD (Fig 2B).

We proposed polydopamine-mediated adsorption of BSA as a simple strategy to improve the colloidal stability of gold nanorod suspensions while minimizing impact to the secondary structure of the protein coating. We found that BSA-PD-NRs were capable of maintaining a higher α-helical propensity (p(α) = 0.899) compared to BSA-only modified counterparts (p(α) = 0.824), and that this ratio more closely approached the control BSA standard (0.25 mg/mL, p(α) = 0.917). The high alpha-helical propensity is considered advantageous, as denaturation may lead to the undesired exposure of immunostimulatory domains in BSA [42, 63]. Furthermore, an electrostatically driven modification as would be the case in the absence of PD, may be unstable in physiological environments [11], permitting the displacement of passivating BSA by higher affinity proteins that can drive the elimination of nanoparticles via the reticuloendothelial system [64]. Partial BSA denaturation may reveal and perturb interior disulfide bonds, which have been shown to contribute to bonding through Au-thiol interactions [65].

Strikingly, BSA-PD-NRs prepared at sub-optimal 10 mg/mL BSA concentrations aggregated as denatured β-sheets (Fig 2B). Hydrogen bonding interactions can bring together antiparallel peptide strands in neighboring BSA molecules, producing characteristic β-sheet CD spectra. At low BSA concentrations, polydopamine may be contributing to intermolecular hydrogen bonding, whereas an optimized excess of BSA may inhibit this bridging. In this case, incomplete BSA passivation may permit PD to contribute to the observed change in secondary structure. Furthermore, high surface coverage density is generally associated with the preservation of protein native structure [66].

### Physiological stability of functionalized AuNRs

To test the ability of modified AuNRs to resist aggregation in physiologically relevant environments, BSA-PD-NR, BSA-NR, PD-NR, and CTAB-NR samples were repeatedly centrifuged and resuspended in DMEM cell culture media and UV-Vis-NIR spectra acquired (Fig D in S1 File). BSA-PD-NRs (20 mg/mL BSA, 0.1 mg/mL PD) demonstrated excellent longitudinal LSPR peak intensity after 5 sedimentation challenges, whereas BSA-NR samples showed a 12% greater loss. The LSPR peak of BSA-PD-NR blue shifted ~5 nm whereas BSA-NR decreased by ~7 nm, suggesting decreased surface stability in the absence of PD. PD-NRs showed evidence of aggregation, as demonstrated by peak broadening and the absence of the longitudinal LSPR peak after 5 cycles. Long-term stability was evaluated over the course of one month, with no appreciable loss in LSPR intensity of BSA-PD-NRs (Fig D in S1 File), underscoring the physiological stability of the modified AuNRs.

**Fig 3:**
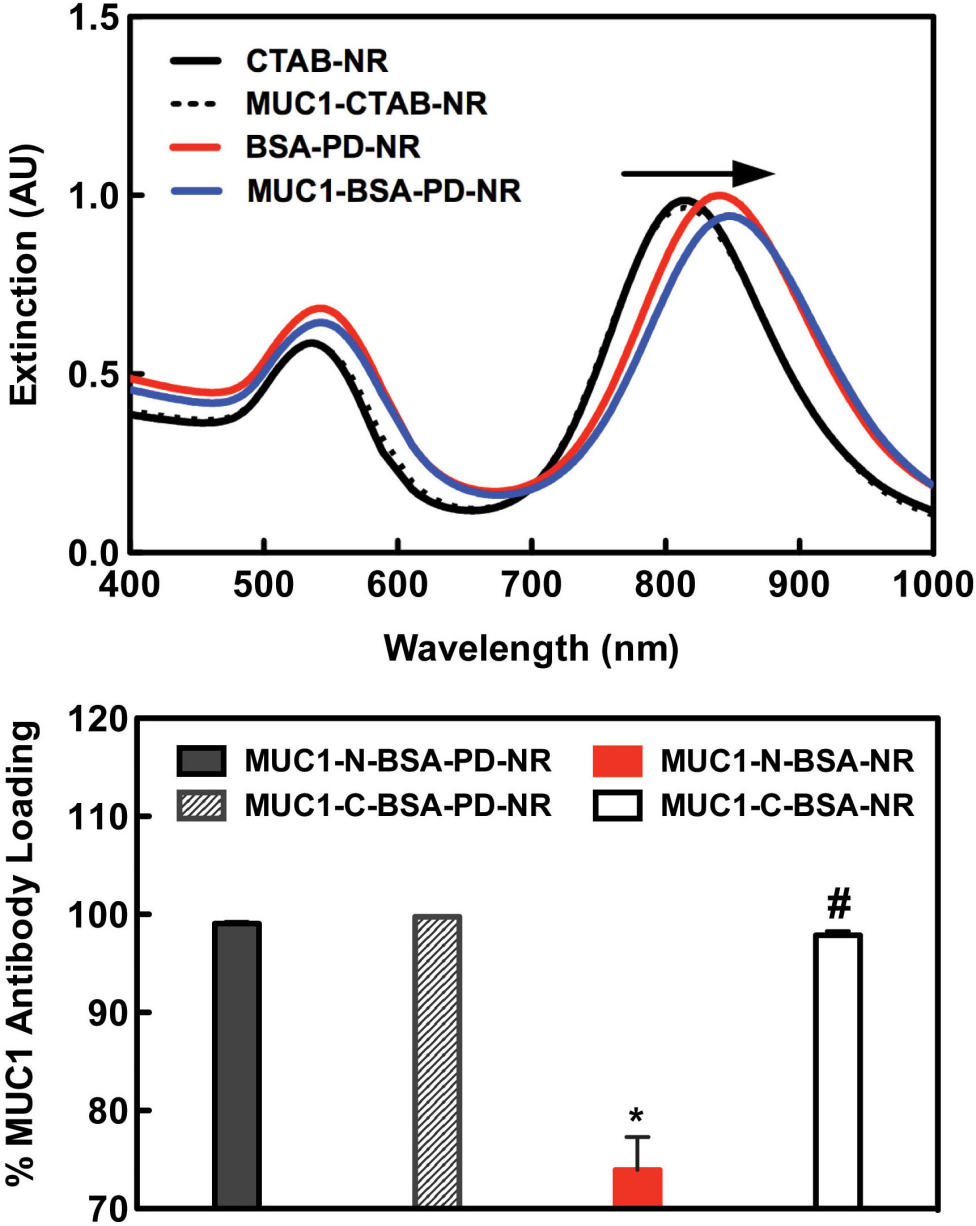
Anti-MUC1 conjugation efficiency. (A) LSPR peak red shifting is observed with sequential BSA and anti-MUC1 modifications. (B) ELISA reveals total incorporation of MUC1-N and—C antibodies was achieved (* p < 0.05) only in PD-primed NRs additionally stabilized via a BSA layer. Near 100% loading in MUC1-C-BSA-NR samples has no practical significance as antibody addition induced complete NR aggregation (#). To quantify the differential MUC1 expression profiles in our cell lines, cells were treated with PE-conjugated MUC1 antibodies and analyzed via flow cytometry for their mean fluorescence intensities (MFI) against cells labeled with isotype controls. Both MCF-7 (MUC1^++^) and SCC15 (MUC1^+^) cells demonstrated an enhanced MFI in agreement with literature reports [16, 67, 68]. As expected, MUC1-deficient MDA-MB-231 (MUC1^-^) cells did not show an appreciable increase in anti-MUC1 PE labeling (Fig F in S1 File).

Antibody conjugation was qualitatively confirmed via SDS-PAGE (Fig E in S1 File) and optical spectroscopy of LSPR red shift (Fig 3A). To quantify the degree of MUC1 antibody conjugation to modified AuNRs, a MUC1 ELISA was designed. Goat anti-mouse coated microplates were used to capture unbound mouse anti-human MUC1 antibodies sourced from AuNR sample supernatants to calculate the amount of depleted antibody from known starting concentrations. The results of the ELISA indicate that antibody was completely incorporated in BSA-PD-NR samples whereas BSA-NR modified with MUC1-N (BSA-NR N) incorporated only 72% of the available antibody under the same conditions (p < 0.05)–affording a facile and versatile coupling strategy for cancer targeting applications. Treatment of BSA-NR with MUC1-C antibodies (BSA-NR C) resulted in the immediate aggregation of the sample (Fig 3B), further supporting the potential covalent nature of the BSA-PD coating and its robust ability to survive in physiologically relevant environments.

### Cytotoxicity and imaging of modified AuNRs in cells

Because cationic CTAB-coated surfaces have been shown to display indiscriminate cytotoxicity by binding to negatively-charged cell membranes [6, 69], we first investigated cytotoxicity of our modified NRs as well as the CTAB-NRs they were prepared from. After modification with PD and BSA, CTAB-mediated cytotoxicity of AuNRs was successfully eliminated (Fig G in S1 File) in accordance with the lack of a positive surface charge (Table 1).

**Fig 4:**
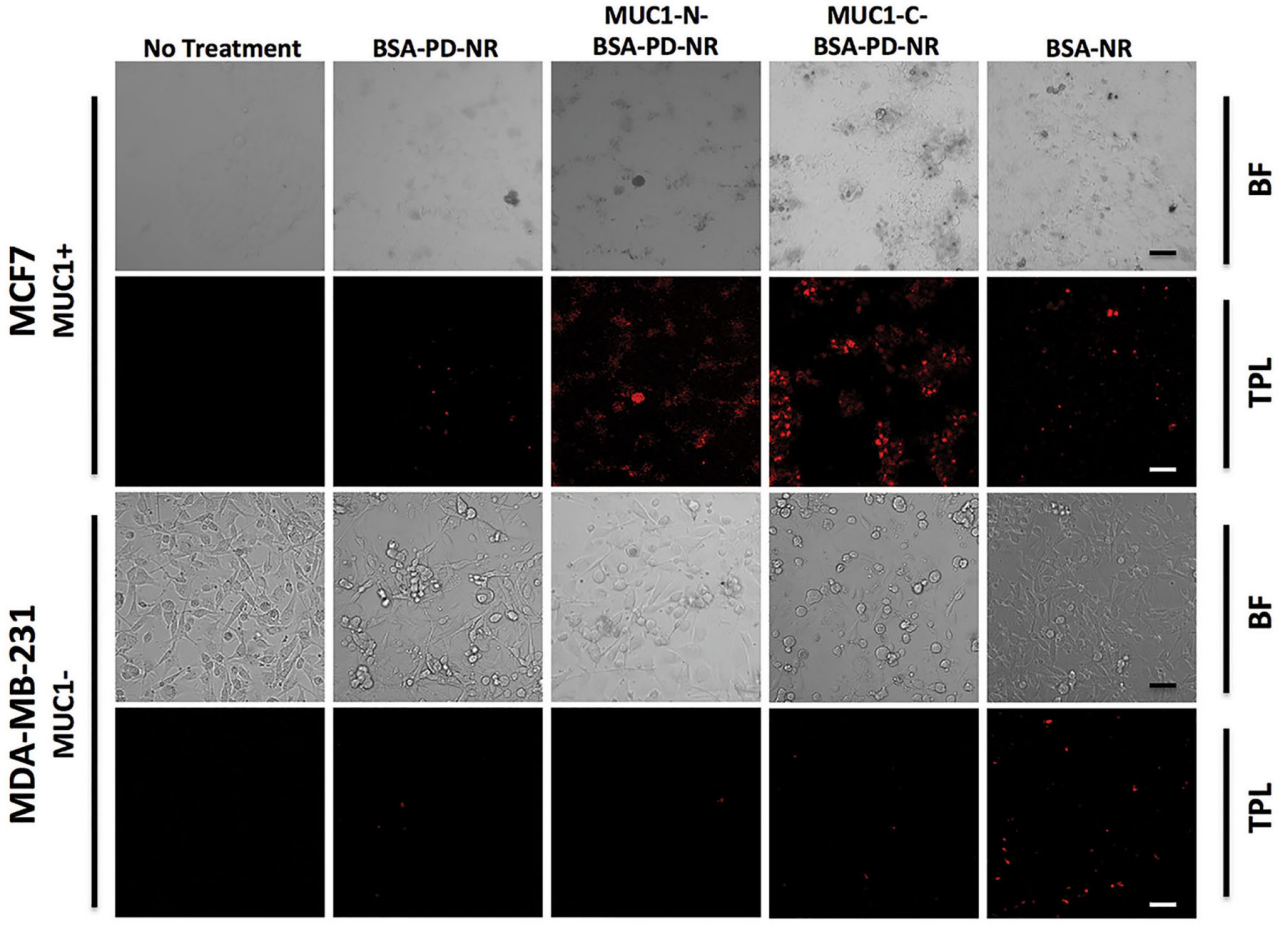
Two-photon luminescence imaging. Two-photon luminescence (TPL) confocal imaging of MCF-7 and MDA-MB-231 breast cancer cells 24 h post treatment with NRs. Brightfield (BF, grayscale) and AuNR TPL images (red) reveal specific targeting in MUC1^+^ MCF-7 cells. Scale bar = 50 μm.

**Fig 5:**
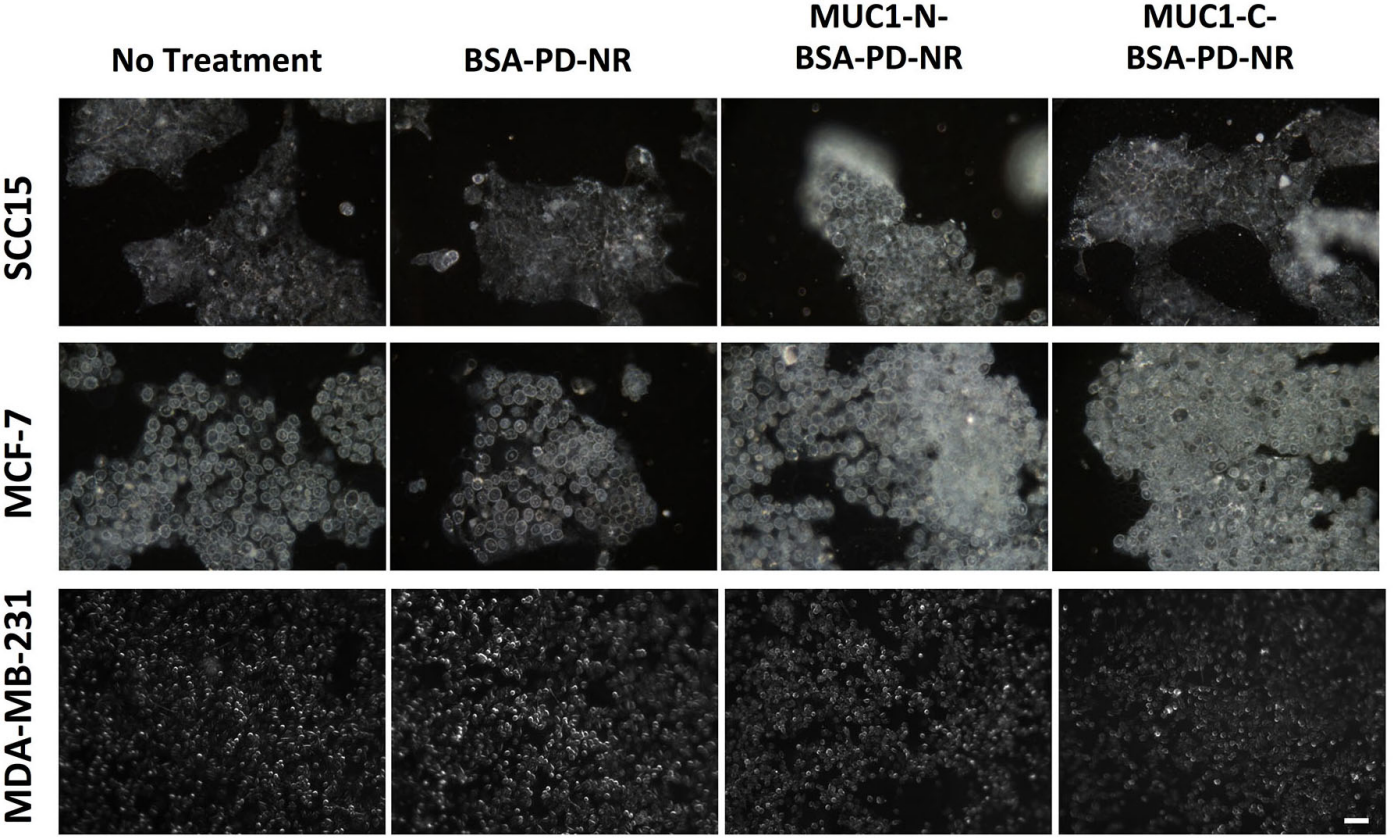
Darkfield imaging. AuNR uptake by cells detected by darkfield scattering imaging of MUC1-overexpressing SCC15 oral or MCF-7 breast cancer cells following treatment with MUC1-modified NRs. Scale bar = 50 μm.

Two-photon luminescence (TPL) imaging of MCF-7 breast cancer cells was pursued following treatment with 3 pM AuNRs (Fig 4). No non-specific cell uptake was observed with BSA-PD-NR, in contrast to BSA-NR. Additionally, enhanced cell colocalization was observed with treatment of MUC1-N- and MUC1-C BSA-PD-NR conjugates. In contrast, imaging of MUC1-deficient MDA-MB-231 breast cancer cells demonstrated no targeting of MUC1-conjugated BSA-PD-NRs and some non-specific uptake of BSA-NRs. In addition to the excellent analytical contrast afforded by TPL imaging, AuNRs are efficient scatterers of incident light, a phenomenon that can be exploited for contrast imaging via darkfield microscopy. Both MCF-7 and SCC15 cells demonstrate enhanced labeling with MUC1-modified BSA-PD-NRs vs. unmodified BSA-PD-NRs (Fig 5). In contrast, some uptake of BSA-NRs was detected in all cell lines regardless of MUC1 status, suggesting that the passivation of the NR surface in the absence of PD is incomplete or is susceptible to displacement.

### In vitro MUC1-BSA-PD-NR-mediated photothermal therapy

Patients with undefined tumor margins or advanced disease may be poor candidates for surgical resection and could benefit from improved multimodal nanotherapeutic approaches [1]. Upon mild heating, enhancement of tissue perfusion and reoxygenation has been shown to synergistically increase the efficacy of chemotherapy and radiation. With the added conjugation of targeting ligands to a nanorod surface, researchers have demonstrated highly desirable ablative specificity with minimal damage to healthy neighboring tissues [4, 5]. The non-invasive penetration of NIR energy to AuNR-treated tissues permits localized tumor ablation via hyperthermia as excited electrons convert adsorbed photon energy into heat [70]. NIR light has been demonstrated to penetrate breast tissue to a depth of at least 10 cm, providing compelling support for future clinical translation [71].

**Fig 6:**
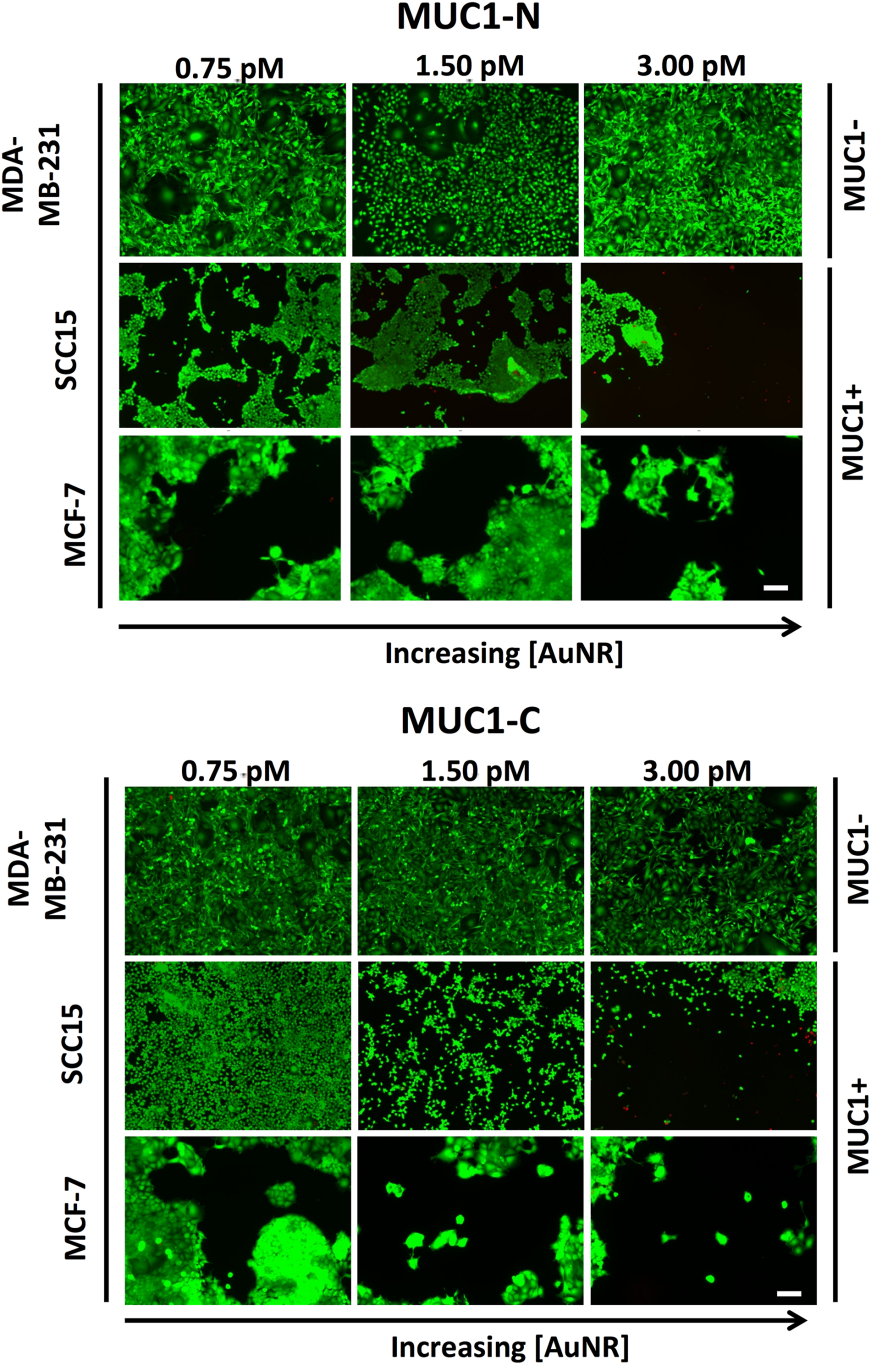
Photothermal therapy of breast and oral cancer cells. Photoablation of cells with NKT Photonics SuperK Versa near-infrared (NIR) light source (480–850 nm). Cells were imaged with calcein AM (live, green) and propidium iodide (dead, red) to visualize photoablation efficiency following treatment with increasing doses of AuNRs. Due to the delamination of dead cells, no red staining is present in the treated regions of interest. In the MCF-7 cell line, this dead staining was occasionally observed prior to delamination (Fig I in S1 File). Scale bar = 100 μm.

Photothermal therapy was evaluated in breast and oral cancer cell lines with varying levels of carcinoma-associated MUC1 expression. MCF-7, MDA-MB-231, and SCC15 cells were treated with 0, 0.75, 1.5, or 3 pM of variously modified AuNRs and subjected to NIR photo-irradiation at a power density of 0.18 W/nm. Fluorescent live/dead staining of cells treated with anti-MUC1-modified AuNRs demonstrated a dose-dependent enhancement of cellular ablation that was MUC1 specific in MCF-7 and SCC15 cell lines (Fig 6). Thermal elevation curves measured in aqueous suspensions of variously modified nanorods revealed heat increases sufficient for therapeutic hyperthermia (Fig H in S1 File).

**Fig 7:**
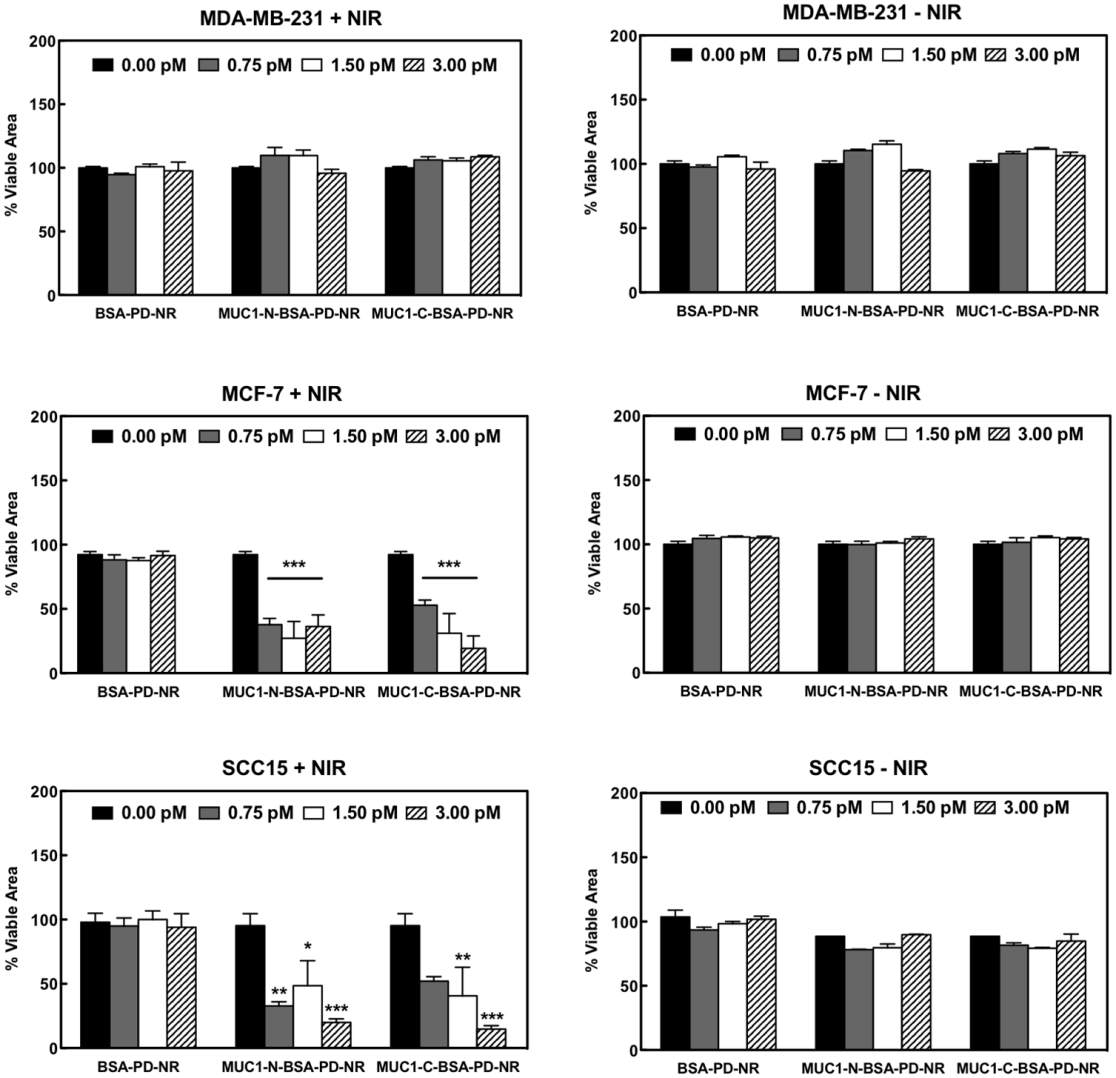
Quantification of breast and oral cancer photothermal therapy. Analysis of normalized viable cell areas reveals MUC1-dependent therapy vs. non-targeted BSA-PD-NRs in MCF-7 and SCC15 cell lines (* p < 0.05, ** p < 0.01, *** p < 0.001). Two-way ANOVA reveals a statistically significant impact of dose (p < 0.0001) and treatment (p < 0.0001). The interaction of dose and treatment was found to be statistically significant only for MCF-7 cells (p < 0.001).

The overall impact on MCF-7 cells was most profound, in accordance with the high carcinoma-associated MUC1 expression observed via flow cytometry. Treatment with MUC1-C-BSA-PD-NR followed similar trends. Because cell death was accompanied by delamination under the conditions of our experiments (Fig I in S1 File), analysis of normalized viable cell areas was used for quantification, revealing MUC1-dependent cell death in anti-MUC1 modified AuNRs vs. non-targeted BSA-PD-NRs in MCF-7 and SCC15 cell lines (* p < 0.001). Two-way ANOVA indicates a statistically significant impact of dose (p < 0.0001) and treatment (p < 0.0001). Photoablation was observed at lower doses in MCF-7 vs. SCC15 cells (Fig G in S1 File), a result that is consistent with their differential MUC1 expression profiles (Fig F in S1 File). MUC1-deficient MDA-MB-231 cell lines did not present any pattern of photoablation upon NIR exposure, nor was viability negatively impacted in non-photoirradiated control samples (Fig 7).

### Conclusions

In this study, we report a method for polydopamine-mediated coating of AuNRs with BSA for passivation and straightforward conjugation of MUC1 antibodies for cancer cell targeting. The modified AuNRs were stable in physiologically relevant culture media and selectively bound to cells overexpressing MUC1. Illumination of treated cells with NIR light induced cell death in MUC1 positive cancer cell lines. Taken together, these results demonstrate the viability of our strategy for the surface modification of AuNRs for cancer cell targeting. Given the emerging body of literature implicating underglycosylated MUC1 in a large number of cancers, the results establish the potential use of MUC1 antibody modified AuNRs for the targeting and photoablation of MUC1-overexpressing epithelial malignancies.

## Supporting Information

S1 File **Fig A) Stepwise modification of AuNR surfaces by polydopamine and BSA induces a shift in the plasmon absorbance**. Normalized LSPR spectra obtained via UV-vis-NIR spectroscopy indicate a red shift in peak position upon surface modification with PD (0.1 mg/mL) and BSA (20 mg/mL). See also [57]. **Fig B) Polydopamine enhances the stability of BSA modified NRs after five cycles of centrifugation and resuspension**. Au-NRs were incubated in BSA at the concentrations shown, subjected to five cycles of centrifugation and redispersion in water and then analyzed for LSPR peak intensity and peak shift. (A) Normalized LSPR peak intensities of PD-NRs treated with 0–30 mg/mL BSA (*** p < 0.001). (B) LSPR shifts of AuNRs with (+PD) and without (-PD) polydopamine treated with 0–30 mg/mL BSA. Generally, a high LSPR peak intensity and low LSPR shift is indicative of improved colloidal stability. Although nanorods without PD were effectively passivated by BSA at high concentration, passivation was achieved at a lower BSA concentration in the presence of PD (p < 0.0001). **Fig C) Au4f XPS analysis of surface modification by PD and BSA [72]**. CTAB-treated Au/Ti/Si wafers were subjected to sham (bicine buffer), PD, BSA, and PD+BSA modifications as a model of AuNR surface coating steps. High-resolution Au4f scans reveal decreasing Au substrate intensities with successive modifications. Less substrate is detected with BSA-only modification compared to PD+BSA coatings, suggesting that PD promotes a thinner or more uniform surface coverage. **Fig D) Physiological stability of modified gold nanorods**. AuNR suspensions were measured by UV-Vis-NIR spectroscopy after sedimentation challenges, consisting of five cycles of centrifugation and pellet redispersion in DMEM cell culture media (A) and PBS (B). Stability of MUC1-modified BSA-PD-NRs was maintained over the course of one month. CTAB-NR is not shown at day 30 due to the complete aggregation of the sample. **Fig E) Qualitative antibody immobilization**. SDS-PAGE analysis of standards (BSA, ladder, and AlexaFluor 633-IgG) and supernatants after modification of AuNRs (IgG-BSA-PD-NR, IgG-BSA-NR) indicates that treatment of AuNR surfaces with PD captures more antibody onto the AuNR as seen in the reduction in IgG band intensity of the supernatant for PD treated AuNRs (lane 4) compared to BSA modified AuNR (lane 5). **Fig F) MUC1 status of cell lines analyzed by flow cytometry**. Labeling with PE-MUC1-N antibody and IgG1a isotype control antibody confirms differential MUC1-N expression in the experimental cell lines: MCF-7, MUC1^++^; SCC15, MUC1^+^; MDA-MB-231, MUC1^-^. **Fig G) Cytotoxicity of biofunctionalized AuNRs**. MDA-MB-231 and MCF-7 breast cancer cell lines were exposed to 3 pM CTAB-NR (blue), BSA-NR (green), or BSA-PD-NR (orange) for 30 minutes and compared against unexposed control cells (red). Cells were stained with PI and analyzed via flow cytometry. CTAB-mediated cytotoxicity was mitigated in all modification conditions. **Fig H) Temperature elevation curves for NIR-exposed nanorod solutions**. Aqueous solutions containing 3 pM BSA, anti-MUC1-N, or anti-MUC1-C-modified PD-NRs demonstrated temperature elevations sufficient for therapeutic hyperthermia [8], supporting the photothermal ablation observed in experiments. These bulk heating profiles differ significantly from NIR-exposed water alone (ΔT ≈ 2–3°C, see Fig 3A in [9]). **Fig I**) **Live/dead staining of MCF7 cells in treated and control conditions**. Photoablation of MCF7 cells (MUC1^++^) with NKT Photonics SuperK Versa near-infrared (NIR) light source (650–850 nm) (+IR). Cells were imaged with calcein AM (live, green) and propidium iodide (dead, red) to visualize photoablation efficiency following treatment with increasing doses of modified AuNRs. In this experiment, the red staining of dead cells could be observed prior to cell delamination. Similar detachment or photothermolysis of ablated cells has been reported in the literature [73, 74, 75, 76, 77]. Scale bar = 100 μm.
(ZIP)

S2 File Supplemental methods detailing UV-vis-NIR, XPS, flow cytometry, SDS-PAGE, temperature elevation, and photothermal experiments are provided. (PDF)
