# Supplementary material for: MUC1-Targeted Cancer Cell Photothermal Ablation Using Bioinspired Gold Nanorods
Source: PLoS One. 2015 Jul 6;10(7):e0128756. doi: 10.1371/journal.pone.0128756 (PMC4493038; doi:10.1371/journal.pone.0128756)
Supplement: S2 File — (PDF) [file pone.0128756.s002.pdf]

Supporting Information

Supplemental Methods

Quantification of AuNR Stability

UV-Vis-NIR spectra of modified and unmodified CTAB-NRs were analyzed with a Hitachi U-2010 spectrophotometer (Hitachi City, Japan). Appropriate reference solutions (10 mM bicine buffer or ultrapure deionized water) were used. Scans were performed in 10 mm pathlength cuvettes over a 400-1100 nm spectral range in 1 nm increments. AuNR samples were analyzed immediately after synthesis and after up to five cycles of redispersion and centrifugation in water. Longitudinal peak absorbance intensities were normalized to the initial value.

X-Ray Photoelectron Spectroscopy (XPS)

Gold substrates were prepared by sputtering 100 nm of Au onto Si wafer chips modified with a 15 nm Ti adhesion layer (Au/Ti/Si substrates). Samples were incubated in 1 ml of dopamine (0.1 mg/ml) and/or albumin solutions (20 mg/ml) for 30 minute periods with sonication in sealed 24-well plates. Substrates that were subjected to successive coating (BSA-PD) were transferred to a fresh well with each modification. The cationic surfactant is crucial in the seed-mediated synthesis of AuNRs and its positive charge is expected to strongly affect the deposition of the subsequent polydopamine and albumin protein layers. Therefore, to better represent the conditions during gold nanorod surface modification, Au/Ti/Si substrates were first incubated in 0.2 M cetyltetraammonium bromide (CTAB).

An Omicron ESCALAB XPS (Taunusstein, Germany) equipped with a monochromated 300 W Al Kα (1486.8 eV) X-ray source and 1.5 mm circular spot size under ultrahigh vacuum ($<10^{-8}$ Torr) was used to acquire spectra at a takeoff angle of 45°. Survey scans were acquired at 0.5 eV resolution over a binding energy range of 0-1100 eV. Detailed scans were acquired at 0.05 eV resolution for C 1s, O 1s, N 1s, and Au 4f emissions. Spectra were calibrated to the C 1s (C-C) peak at 284.6 eV.

Flow Cytometry

MCF7 and MDA-MB-231 cells were grown to confluence and harvested via trypsinization. Cells were rinsed and resuspended at $1x10^6$ cells/ml in media. The cell suspension (400 μl) was aliquoted and mixed with 100 μl of modified AuNR solutions prepared by pellet dilution in 2 ml PBS after three centrifugation steps. Propidium iodide was added to each sample to achieve a final concentration of 2 μg/ml. Samples were allowed to incubate at 37°C for 30 minutes prior to acquisition with a BD LSRII flow cytometer (BD Biosciences, San Jose, CA). A minimum of 20,000 events were collected and gated to exclude debris. To confirm MUC1 status, MCF7, MDA-MB-231, and SCC15 cells were harvested, fixed in 4% paraformaldehyde, incubated with PE-conjugated MUC1-N antibodies, washed, and analyzed for shifts in mean fluorescence intensities compared to unlabeled Isotype controlled counterparts. Data was processed with BD FACSDiva (San Jose, CA) and FlowJo (Ashland, OR) software suites.

Gel Electrophoresis

Antibody binding was assessed via sodium dodecyl sulfate polyacrylamide gel electrophoresis (SDS-PAGE). Supernatants were separated from BSA-PD-NRs and BSA-NRs

treated with 0.4 μg IgG-AlexaFluor 633 via centrifugation. Supernatants were mixed to a final concentration of 2.5% sample and 50% Laemmli sample buffer and heated at 95°C for 5 minutes. Antibody losses in sample supernatants were identified by electrophoresis with a separating gel containing a 4-15% Tris-HCl polyacrylamide gradient. Gels were stained with Sypro Ruby and imaged with a Typhoon 94000 fluorimager (GE Healthcare).

Temperature Measurements

Aqueous solutions containing BSA-PD-NR, MUC1-N-BSA-PD-NR, or MUC1-C-BSA-PD-NRs (3 pM) were illuminated with a SuperK Versa broadband laser source (NKT Photonics, 480-850 nm, 1 mm spot) for 3.5 min at a power density of 0.18 W/nm at the plasmon center wavelength (780 nm). Temperature changes were measured during exposure with an I652 Industrial Fluoroptic Thermometer (Luxtron, Santa Clara, CA).
